# A Comprehensive Roadmap Towards the Generation of an Influenza B Reporter Assay Using a Single DNA Polymerase-Based Cloning of the Reporter RNA Construct

**DOI:** 10.3389/fmicb.2022.868367

**Published:** 2022-05-25

**Authors:** Nandita Kedia, Saptarshi Banerjee, Arindam Mondal

**Affiliations:** School of Bioscience, Indian Institute of Technology Kharagpur, Kharagpur, India

**Keywords:** influenza B, reporter construct, ribonucleoprotein, polymerase activity assay, antiviral screening

## Abstract

The mini-genome reporter assay is a key tool for conducting RNA virus research. However, procedural complications and the lack of adequate literature pose a major challenge in developing these assay systems. Here, we present a novel, yet generic and simple, cloning strategy for the construction of an influenza B virus reporter RNA template and describe an extensive standardization of the reporter RNP/polymerase activity assay for monitoring viral RNA synthesis in an infection-free setting. Using this assay system, we showed for the first time the effect of viral protein NS1 and host protein kinase C delta (PKCD) on influenza B virus RNA synthesis. In addition, the assay system showed promising results in evaluating the efficacy of antiviral drugs targeting viral RNA synthesis and virus propagation. Together, this work offers a detailed protocol for the standardization of the influenza virus minigenome assay and an excellent tool for screening of host factors and antivirals in a fast, user-friendly, and high-throughput manner.

## Introduction

First discovered in 1940 (Francis, 1940), the influenza B virus has since caused significant morbidity and mortality in the global population (Sharma et al., 2019). As per the recent surveillance (2010–2018 seasons), influenza B viruses are responsible for 15–30% of total influenza-like illness, with various complications such as fever, body aches, fatigue, and even life-threatening acute respiratory distress syndrome in patients with pre-existing lung diseases (CDC, 2021a,b). There are two different lineages of influenza B virus, Victoria and Yamagata. They circulate in the human population with varying degrees of predominance in different influenza seasons (McCullers et al., 2004; Xu et al., 2015). Due to the constant increase in influenza B virus-related infections and the limited cross-protection offered by the influenza B vaccine against both lineages, there is a gradual transition from trivalent (against two subtypes of Flu A and one lineage of Flu B) to quadrivalent (against two subtypes of Flu A and two lineages of Flu B) flu shots being offered worldwide (Ambrose and Levin, 2012). In spite of its immense importance in the context of global healthcare ecosystem, research on influenza B virus has drawn significantly less attention compared to the closely related influenza A viruses, largely due to the scarcity of tools required to study the virus replication cycle. This also severely restricts antiviral drug discovery directed toward influenza B virus therapy (Dumm and Heaton, 2019).

Influenza viruses are segmented negative-stranded RNA viruses of the family Orthomyxoviridae. Among the four types A, B, C, and D, only influenza A and B cause human epidemics (Krammer et al., 2018). The viral genome consists of eight different segments, each of which remains enwrapped with multiple copies of nucleoprotein (NP) in its oligomeric form and associates with a single copy of RNA-dependent RNA polymerase (RdRp) to form ribonucleoprotein complexes or RNPs (Bouvier and Palese, 2008). RNPs are the self-sufficient machinery for driving different modes of RNA-dependent RNA synthesis events, including viral gene expression and genome replication, hence reside at the center of the virus replication cycle (Fan et al., 2019). Therefore, reporter RNP-based assay systems remain one of the invaluable tools for studying virus replication, host–pathogen interaction, and high-throughput screening of antivirals without handling infectious virus particles and thus avoiding biosafety-associated procedural complications (Lutz et al., 2005; Zhu et al., 2011).

Influenza virus genomic segments are single-stranded RNAs that are devoid of the 5′-Caps and 3′-Poly(A) tails (Dadonaite et al., 2019). Different segments harbor conserved untranslated regions (UTRs) of varying lengths at both the 5′ and 3′ ends, which bracket single or multiple open reading frames (in the antisense orientation) encoding viral proteins (Szymkowiak et al., 2003; Dadonaite et al., 2019). The terminal regions of the 5′ and 3′ UTRs contain complementarity that results in a partial duplex structure (also known as “panhandle RNA” or “cork-screw RNA”) that serves as a promoter of viral RdRp (Flick and Hobom, 1999). Additionally, UTRs contain cis-acting elements, necessary and sufficient for the transcription and replication of viral (Park and Katze, 1995; Fodor et al., 1998; Ferhadian et al., 2018) and non-viral reporter genes (Neumann et al., 1994; Flick and Hobom, 1999; Lutz et al., 2005; Sherry et al., 2014). Several groups have established reporter RNA-based assay systems in which viral open reading frames have been replaced by reporter genes of fluorescent, bioluminescent, or chemiluminescent proteins (Luytjes et al., 1989; Parvin et al., 1989; Flick and Hobom, 1999; Lutz et al., 2005; Wang and Duke, 2007; Li et al., 2009; Zhu et al., 2011). These reporter RNA templates, when expressed inside the cells in combination with NP and RdRp proteins, reconstitute reporter RNPs. The RNA synthesis activity of these RNPs could be measured by quantifying the extent of reporter gene expression. Although this appears a straightforward procedure, but the successful establishment of a reporter assay system requires (i) complicated cloning strategies to synthesize the reporter RNA construct, (ii) the construction of plasmids for the expression of viral NP and RdRp subunits (PB1, PB2, and PA) and (iii) optimized expression of the reporter RNA and viral proteins in required stoichiometric amounts that lead to the reconstruction of reporter RNPs with maximum efficiency.

So far, different strategies have been used to construct plasmids expressing the influenza A and B virus reporter RNA template (reporter plasmid) (Lutz et al., 2005; Wang and Duke, 2007; Li et al., 2009; Zhu et al., 2011; Moncorge et al., 2013). In a few studies, the reporter luciferase gene was amplified using primers containing long overhangs corresponding to 3′ and 5′ UTR regions of influenza A or B viruses; then, the resulting polymerase chain reaction (PCR) fragment harboring the luciferase open reading frame (ORF) flanked by the viral UTRs was inserted into the target vector for RNA polymerase-I-driven expression of the same, using conventional restriction digestion and subsequent ligation method (Lutz et al., 2005; Wakai et al., 2011; Sherry et al., 2014). Alternatively, viral vectors containing 5′ and 3′ UTRs (amplified using inverse PCR from the cDNA clone of the corresponding segment) were ligated with the reporter gene insert predigested with compatible restriction enzyme sites (Muramoto et al., 2006). In another cloning strategy, a double-stranded DNA linker encompassing 5′ and 3′ UTRs was inserted into the vector between the Pol-I promoter and terminator sequences with the help of compatible restriction enzyme sites. The reporter gene was then inserted between the UTRs using a second restriction enzyme site (Li et al., 2009). All of these restriction enzyme-based cloning strategies are laborious and often introduce additional nucleotides between the UTRs and the reporter gene, which may interfere with the activity of the cis/trans acting elements (Li et al., 2009). To avoid these constraints, restriction enzyme-free cloning methods utilizing vectors and inserts containing overlapping sequences have also been implemented. For example, inserts containing reporter genes flanked by 5′ and 3′ UTRs were created using long overhang primers (containing the UTR regions), which were then stitched to the vector through the use of proprietary specialized enzymes/kits (Zhu et al., 2011; Zhang et al., 2020). With the inherent limitations of the aforesaid cloning techniques, the scarcity of information on the extensive experimental protocol makes it difficult to establish and standardize the reporter-based RNP activity assay for influenza viruses. This situation is further complicated for influenza B viruses due to the larger size of UTRs compared to those in influenza A viruses.

Here, we present a novel yet fairly simple cloning strategy, independent of any restriction enzyme or specialized reagents or kits, to construct a firefly luciferase-based reporter plasmid capable of generating a reporter genome template for influenza B/Brisbane/60/2008 virus. Additionally, we present an extensive standardization of this reporter plasmid-based RNP activity assay through the optimization of various parameters that regulate viral RNA synthesis. Using the reporter assay system, we have shown for the first time the effect of viral non-structural protein-1 (NS1) and host protein kinase C delta (PKCD) upon influenza B virus RNA synthesis. We have also demonstrated the ability of this assay system to be used as a high-throughput screening platform for the identification of antiviral drugs that specifically inhibit viral RNA polymerase activity. Together, this work presents a great resource for the cloning, standardization, and implementation of the reporter-based RNP activity assay for influenza and other related viruses.

## Materials and Methods

### Cell Lines and Viruses

Human embryonic kidney 293T (HEK 293T) cells were maintained in Dulbecco's modified Eagle's medium (DMEM; Gibco™, Cat no. #12800017) supplemented with 10% (v/v) fetal bovine serum (FBS; Gibco™, Cat no. #10082147), 2 mM GlutaMAX™ (Gibco™, Cat no. #35050061), 1% penicillin–streptomycin (Gibco™, Cat no. #1514122), incubated at 37°C in a humidified 5% CO_2_ incubator. Madin-Darby Canine Kidney (MDCK) cells were maintained under the same conditions with 10% FBS (Gibco™ Cat no. #10270106). Influenza B/Brisbane/60/2008 virus was used in this study.

### Virus Amplification and RNA Extraction

About 3 × 10^6^ MDCK cells were seeded in 10-cm dishes, 24 h before infection. Prior to infection, the cell monolayer was washed with phosphate-buffered saline (PBS) two times and subsequently infected at an M.O.I. of 0.001. For each 10-cm dish, 1 ml of virus inoculum was prepared in viral growth media [VGM; containing DMEM, 0.2% bovine serum albumin (Sigma; Cat no. #A8412), 25 mM N-(2-hydroxyethyl) piperazine-N′-ethanesulfonic acid (HEPES; Invitrogen, Cat no. #15630080) buffer, 2 mM GlutaMAX™, 1% penicillin–streptomycin, and 0.5 μg/ml TPCK-trypsin (Thermo Scientific™, Cat no. #20233)]. Virus attachment was performed with 1 ml of inoculum for 1 h at 37°C in humidified 5% CO_2_ incubator with intermittent shaking at every 10 min to prevent drying of cell monolayer and homogenous distribution of the inoculum. Post attachment, each 10-cm dish was supplemented with 6 ml of VGM and incubated at 33°C in a humidified 5% CO_2_ incubator. At 72 h postinfection, the supernatant were collected and centrifuged at 3,200 g for 10 min at 4°C to remove cell debris. Supernatants were collected, and aliquots were stored in a refrigerator at 80°C for further applications (Eisfeld et al., 2014).

### Reverse Transcription PCR

Viral RNA was extracted from the amplified virus stock using the Trizol reagent (Invitrogen; Cat no. #15596018). Reverse transcription was carried out using the in-house produced Moloney murine leukemia virus (MMLV) Reverse transcriptase (RT) enzyme and the primer “Uni9,” which is complementary to all the segments of influenza B/Brisbane/60/2008 virus. The sequences of the primers are mentioned in Table 1. Briefly, 10 μl of viral RNA (~500 ng), 1 μl of 2 μM Uni9 primer, and 1 μl of 10 mM dNTP (Thermo Scientific™, Cat no. # R0181) were mixed and warmed at 65°C for 5 min. This is followed by snap chilling at ice for 2 min. After snap chilling of the reaction, the premixed solution containing 4 μl of 5 × RT buffer (Invitrogen, Cat no. #18080044), 2 μl of 0.1 M DTT (kit component of Invitrogen, Cat no. #18080044), 0.25 μl of RNase inhibitor (Thermo Scientific™, Cat no. #EO0381), 1 μl of MMLV RT and 0.75 μl of sterile nuclease-free water (AMRESCO, Cat no. # E476-1L) were added to the tube containing the snap chilled mix. The reaction was carried out at 42°C for 50 min and terminated by heating at 70°C for 15 min. After the RT reaction, in which the cDNA for all eight segments was generated; the PCR reaction was carried out using NA-NB segment specific primer to enrich the fragment specific for segment six using the primers “NA-NB_F” and “NA-NB_R” (primer sequences are listed in Table 1). Each of 50 μl of PCR reaction mixture consisted of 10 μl of 5 × Phusion HF buffer, 5 μl each of 5 μM primers, 5 μl of 2 mM dNTPs, 5 μl each of the synthesized cDNAs, 19.5 μl of sterile nuclease-free water, and 0.5 μl of Phusion high-fidelity DNA polymerase (Thermo Scientific™, Cat no. #F530S). Cycling conditions for the PCR reaction are stated in Table 2. For RT reactions, lyophilized primers were dissolved and diluted in sterile nuclease-free water. For PCR-specific primers, initial stocks were dissolved with 10 mM Tris-HCl (pH = 8) and working stocks were diluted in sterile nuclease-free water. PCR products were purified using PCR Purification kit (Invitrogen; Cat no. # K310001). The yield and quality of the purified products were checked by measuring the UV absorbance at 260 nm and 280 nm, and subsequently running them on agarose gel electrophoresis.

**Table 1:** Primers used in this study.

**Name of the primer**	**Sequence (5^**′**^->3^**′**^)**
Uni 9	AGCAGAAGC
NA-NB_F	AGTAGTAACAAGAGCATTTTTCAG
NA-NB_R	AGCAGAAGCAGAGCATC
5′ UTR_F	CATTTTGGGCCGCCGGGTTATTAGTAGTAACAAGAGCATTTTTCAG
5′ UTR_R	CGGAAAGATCGCCGTGTAATGGAGGAATGGTTGAGTC
3′ UTR_F	CTTTATGTTTTTGGCGTCTTCCATTGTTCATTTTTGGCCTATTTG
3′ UTR_R	CCTCCGAAGTTGGGGGGGAGCAGAAGCAGAGCATCTTC
PHH21_F	CCCCCCCAACTTCGGAGG
PHH21_R	AATAACCCGGCGGCCCAAAATG
PHH21 SEQ2	AAAACGCTGGGCGTTAATCAAAGAGGCG
PHH21 SEQ1	GGGGGACACTTTCGGACATCTGGTC
pcDNA3-V5 For	GATCCGGAGGTAAGCCTATCCCTAACCCTCTCCTCGGTCTCGATTCTACGTAGTAAGC
pcDNA3-V5 Rev	GGCCGCTTACTACGTAGAATCGAGACCGAGGAGAGGGTTAGGGATAGGCTTACCTCCG
NP_For	ATTCGGGGTACCGCCACCATGTCCAACATGGATATTGACG
NP_Rev_V5	ATTCGCGGATCCACCATAATCGAGGTCATCATAATCCTC
NP_Rev_Stop	ATTCGCGGATCCTTAATAATCGAGGTCATCATAATCCTC
PB1_For	TAAGCGGAATTCACCATGAATATAAATCCTTATTTTCTCTTC
PB1_Rev_FLAG	ATTGAGGCGGCCGCTATGTACCCAATCTCACCAAG
PB1_Rev_Stop	ATTGAGGCGGCCGCTTATATGTACCCAATCTCACC
PB2_For	TAAGCGGAATTCACCATGACATTGGCCAAAATTGAATTG
PB2_Rev_FLAG	ATTGAGGCGGCCGCGCTCAAGGCCCACCCC
PB2_Rev_Stop	ATTGAGGCGGCCGCTTAGCTCAAGGCCCACCC
PA_For	TAAGCGGAATTCACCATGGATACTTTTATTACAAGAAACT
PA_Rev_FLAG	ATTGAGGCGGCCGCTTCGTCCATAATCTCGTC
PA_Rev_Stop	ATTGAGGCGGCCGCTTATTCGTCCATAATCTCGTC

**Table 2:** PCR conditions used in the study for amplification of individual inserts.

**PCR amplification**	**Forward primer**	**Reverse primer**	**Denaturation (temp./duration)**	**Cyclic denaturation, annealing and elongation (temp./duration)**	**No. of cycles**	**Final elongation**
NA-NB fragment	NA-NB_F	NA-NB_R	98°C/30 s	98°C/10 s 60°C/30 s 72°C/95 s	35	72°C/5 min
5′ UTR	5′ UTR_F	5′ UTR_R	98°C/30 s	98°C/10 s 60°C/25 s 72°C/5 s	35	72°C/5 min
3′ UTR	3′ UTR_F	3′ UTR_R	98°C/30 s	98°C/10 s 59°C/25 s 72°C/5 s	35	72°C/5 min
Insert for Reporter plasmid	5′ UTR double-stranded PCR product	3′ UTR double-stranded PCR product	98°C/30 s	98°C/10 s 63°C/30 s 72°C/80 s	35	72°C/5 min
Vector for Reporter plasmid	PHH21_F	PHH21_R	98°C/30 s	98°C/10 s 67°C/30 s 72°C/90 s	35	72°C/5 min
PB1-FLAG	PB1_For	PB1_Rev_FLAG	98°C/30 s	98°C/10 s 60°C/30 s 72°C/90 s	35	72°C/10 min
PB1-STOP	PB1_For	PB1_Rev_Stop	98°C/30 s	98°C/10 s 58°C/30 s 72°C/90 s	35	72°C/10 min
PB2-FLAG	PB2_For	PB2_Rev_FLAG	98°C/30 s	98°C/10 s 69°C/30 s 72°C/90 s	35	72°C/10 min
PB2-STOP	PB2_For	PB2_Rev_Stop	98°C/30 s	98°C/10 s 69°C/30 s 72°C/90 s	35	72°C/10 min
PA-FLAG	PA_For	PA_Rev_FLAG	98°C/30 s	98°C/10 s 60°C/30 s 72°C/90 s	35	72°C/10 min
PA-STOP	PA_For	PA_Rev_Stop	98°C/30 s	98°C/10 s 58°C/30 s 72°C/90 s	35	72°C/10 min
NP-V5	NP_For	NP_Rev_V5	98°C/30 s	98°C/10 s 64°C/30 s 72°C/90 s	35	72°C/10 min
NP-STOP	NP_For	NP_Rev_Stop	98°C/30 s	98°C/10 s 66°C/30 s 72°C/90 s	35	72°C/10 min

### PCR and Cloning

#### Amplification of 5′ and 3′ UTRs

The reverse transcription PCR- (RT-PCR-) amplified DNA corresponding to segment six of the viral genome was used as a template for the amplification of 5′ and 3′ UTRs using Phusion high-fidelity DNA polymerase with the pairs of primers 5′ UTR_F, 5′ UTR_R, and 3′ UTR_F, 3′ UTR_R (all the primer sequences are listed in Table 1 and the PCR conditions are listed in Table 2), respectively. The PCR product were purified using the Quick gel extraction kit (Invitrogen: Cat no. # K210012). The purified double-stranded 5′ UTR and 3′ UTR fragments (containing overhangs for the insert and vector fragment) were then used as primers for the amplification of luciferase gene (insert amplification), as described in the next section.

#### Preparation of Insert

The luciferase ORF was amplified using pHH21-vNA-Luc, which was kindly provided by Dr. Andrew Mehle, as a template. This plasmid encodes firefly luciferase gene flanked by Influenza A UTR's. Double-stranded 5′- and 3′-UTR fragments, synthesized in the previous step, were used as primers (5 μM final concentration) for the PCR amplification of Luciferase ORF using Phusion high-fidelity DNA polymerase, following the manufacturer's protocol (for primer sequences and PCR conditions, refer to Tables 1, 2, respectively). The PCR product was analyzed on a 0.8% agarose gel and purified using the PCR purification kit.

#### Preparation of the Vector

The vector was amplified using the pHH21-vNA-Luc as a template. “PHH21_F” & “PHH21_R” primers (listed in Table 1) were used to amplify and linearize the vector using Phusion high-fidelity DNA polymerase using 5 × Phusion GC rich buffer following the manufacturer's protocol. Primer sequences and PCR conditions are listed in Tables 1, 2, respectively. The PCR product was analyzed on a 0.8% agarose gel and purified using the PCR purification kit.

#### Circular Polymerase Extension Cloning

In the final circular polymerase extension cloning (CPEC) assembly and cloning reaction (Quan and Tian, 2011), four different CPEC reactions have been set up using the purified linearized vector (as mentioned in Section Preparation of the Vector) and inserts (as mentioned in Section Preparation of Insert) maintaining a molar ratio (V:I) = 1:0, 1:1, 1:2, and 1:3, respectively (**Figure 2D**). The reaction mixture composition is described as follows:

**Table:** 

**Initial concentration**	**Volume per 20 μl reaction**	**Final amount per 20 μl reaction**
Phusion HF buffer (5 ×)	4 μl	1 ×
dNTP mix (40 mM)	0.4 μl	0.8 mM
Phusion high-Fidelity DNA polymerase	0.25 μl	0.5 U
Vector DNA	–	100 ng
Insert DNA	Variable	0, 65.4, 130.8 and 196.14 ng (for V:I molar ratio 1:0, 1:1, 1:2, and 1:3)
Nuclease-free water	Upto 20 μl	–

Cycling conditions for the CPEC assembly and cloning reactions are described as follows:

**Table:** 

**Cycle number**	**Denature**	**Anneal and extend**	**Extend**
1	98°C, 30 s		
25	98°C, 10 s	72°C, 90 s	
26			72°C, 10 min

To assess if a CPEC reaction is successful or not, 10 μl of each product was analyzed in agarose gel electrophoresis (**Figure 2D**). The V:I = 1:3 reaction showing the highest intensity of the high molecular weight band (corresponding to the total length of vector and insert) was selected for transformation.

### Transformation

*Escherichia coli*: DH5α competent cells were prepared by the modified rubidium chloride method as described by Glover (1984). For transformation, 10 μl of the CPEC reaction mixture was added to the competent cells. After an incubation period on ice for 30 min, the cells were subjected to a brief heat shock at 42°C for 35 s, followed by 5 min of incubation on ice. 400 μl of Luria bertani broth (Himedia, Cat no. # M1245; 2.5% in double distilled water) were added immediately after incubation and the cells were grown for 1.5 h in an incubator at 37°C with shaking at 220 rpm. Once the shaking period is over, the entire culture volume was spread on LB-agar plates containing 100 μg/ml of ampicillin. Positive clones were identified by colony PCR and confirmed by Sanger's sequencing.

### Generation of Polymerase Protein Expressing Plasmids

PB2, PB1, and PA ORFs were cloned into the pCDNA-3 × -FLAG vector (generously provided by Dr. Andrew Mehle), which is a modified version of the pcDNA3.1 (addgene) vector expressing proteins under the CMV promoter. This vector contains three FLAG epitopes joined in tandem (3 × -FLAG) after the NotI site at its multiple cloning site (MCS) followed by a cytosine. This results in the expression of a protein that has a tri-alanine linker between the individual ORFs and the C-terminal 3 × -FLAG tag. For the expression of the untagged version of each RdRp subunit, the stop codon has been kept intact at the end of the ORF. For the expression of the V5-tagged NP, pcDNA3 vector has been modified to have a glycine–glycine–serine–glycine linker in between the ORF and the C-terminal V5 epitope tag. Briefly, two primers of 58 nucleotide length (“pcDNA3-V5 For” and “pcDNA3-V5 Rev”) were annealed to create double-stranded piece of DNA (the V5 linker) having sticky ends on both the sides (BamHI restriction site at the beginning of the sequence and the NotI site at the end of the sequence). The thermal protocol for ramp down annealing was as follows: 95°C for 5 min, 70 cycles of 95°C (−1 °C/cycle) each for 1 min, followed by a hold at 4°C. The V5 linker was phosphorylated at the 5′ end by treatment with T4 Polynucleotide Kinase (PNK, Cat no. # M0201S). The pcDNA3.1 (addgene) vector was digested with BamHI and NotI, treated with Calf alkaline phosphatase (CIP; NEB, Cat no. #M0290S) and ligated with the V5 linker at a V: I = 1:20 ratio.

Each individual insert fragment has been amplified using the cDNA template with primers containing the restriction enzyme overhangs. PB2, PB1, and PA have been amplified with EcoRI and NotI overhang in two different PCR sets, one omitting the stop codon and the other including the stop codon in the reverse primer. The NP has been amplified using primers with KpnI and BamHI overhangs. For each amplification, 50 μl of PCR reaction consisted of 10 μl of 5 × Phusion HF buffer, 5 μl each of the forward and reverse 5 μM primers, 5 μl of 2 mM dNTPs, 5 μl of cDNA template, 19.5 μl of sterile nuclease-free water, and 0.5 μl of Phusion high-fidelity DNA polymerase. All the primer sequences and the specific PCR conditions are listed in Tables 1, 2, respectively). The modified pcDNA3-3 × -FLAG have been digested with EcoRI and NotI (NEB), and the pcDNA3-V5 vector has been digested with KpnI and BamHI, followed by CIP treatment. The digested vectors as well as the insert fragments were gel excised and ligated at a vector to insert ratio of 1:3 using T4 DNA ligase (Thermo Scientific™, Cat no. # EL0011) as per the manufacturer's protocol. Around 10 μl of the ligation mixture was transformed into DH5α competent cells (as described in Section Transformation, except that 150 μl of culture volume was spread upon LB-agar plates at the end of the transformation). All positive clones were identified by colony PCR and confirmed by Sanger's sequencing.

### Transfection

To examine the protein expression level of each plasmid, HEK 293T cells were transfected using lipofectamine 3000 (Invitrogen: Cat no. # L3000015). Each plasmid was prepared using the plasmid DNA isolation kit (Promega, Cat no. # A1222), and 100 ng of working stocks were prepared. The pcDNA3.1 blank vector was used for control sets. Briefly, 26 μl of the mixture of optiMEM (25 μl, Thermo Scientific™, Cat no. #31985-070) and p3000 (1 μl) were added to 500 ng of plasmid DNA and mixed well. Around 26.5 μl of the mixture of optiMEM (25 μl) and lipofectamine 3000 (1.5 μl) was further added to the DNA-p3000 premixture, mixed well, and incubated for 15 min at room temperature. About 2.5 × 10^5^ HEK293T cells were seeded into 24-well plates, the transfection mixture was added to the respective wells and kept for incubation at 37°C in a humidified CO_2_ incubator. The media was changed 12 h post-transfection and incubated for 36 h (or stated otherwise) following transfection.

### Western Blot

Protein levels of transiently transfected cells were assessed by western blotting. Transfected cells were lysed for 20 min in pre-chilled Co-Immunoprecipitation buffer (50 mM Tris-HCl pH 7.4, 150 mM NaCl, 0.53% NP-40) supplemented with 1 × protease inhibitor (Roche-Sigma, Cat no. # 11873580001) and 1 × phosphatase inhibitor (Thermo Scientific™, Cat no. #78420). Lysates were centrifuged at 21,000 g at 4°C to remove cell debris, the supernatant was collected, mixed with sodium dodecyl sulfate-polyacrylamide gel electrophoresis sample buffer, and boiled for 10 min. Total protein was separated *via* 8% SDS-PAGE and transferred to polyvinylidene difluoride (PVDF, Bio-Rad, Cat no. #1620177) membrane using transfer buffer (25 mM Tris, 191 mM glycine, 0.025% SDS, and 10% methanol (vol/vol) in the Trans-Blot Turbo Transfer System (Bio-Rad). After incubation with 5% non-fat milk in TBST (50 mM Tris, pH 7.4, 150 mM NaCl, 0.5% Tween 20) for 60 min, the membrane was washed one time with TBST and incubated with antibodies against FLAG (1:5,000, Sigma, Cat no.# F3165), V5 (1:5,000, CST, Cat no. # D3H8Q), HA (1:5,000, CST, Cat no. # C29F4), GAPDH (1:5,000, Biobharati LifeScience Pvt. Ltd., Cat no.# BB-AB0060) BNP (1:5,000, generated in collaboration with BioBharati LifeScience Pvt. Ltd., India), at 4°C for 12 h. The membranes were washed with TBST three times for 5 min and incubated with a 1:25,000 dilution of horseradish peroxidase-conjugated anti-mouse (Sigma, Cat no. # A9044-2ML6) or anti-rabbit (Sigma, Cat no. # A0545-1ML) antibodies for 1 h. The blots were washed with TBST three times for 5 min and developed with the ECL system (ThermoFisher, Cat no. #34095) according to the manufacturer's protocols.

### Polymerase Activity Assay

For polymerase activity assay, the sets in which no additional plasmid DNA other than the core RNP components were used (e.g., **Figures 3D,E**, **5C,D**), 2.5 × 10^5^ HEK 293T cells were co-transfected with 118 ng of each of the pcDNA3-PB2-FLAG, pcDNA-PB1, 15 ng of pcDNA-PA, 125 ng of pcDNA3-BNP, and 125 ng of pHH21-BNA-Luc plasmids to reconstitute 500 ng of total DNA (1/4th BNP, 1/4th pHH21-BNA-Luc, and the half of the total amount of DNA have been divided as 8:8:1 ratio for PB2, PB1, and PA). For the negative control set, pcDNA3.1 blank vector was used instead of the PB2 subunit. For the experiment in **Figure 3A**, 100 ng of each polymerase subunit have been transfected, which has been updated in the subsequent experiments. For the polymerase activity assay with additional plasmid components (e.g., **Figures 4A,B**), the total RNP reconstituting plasmids have been reduced to 400 ng by transfecting 94.11 ng of each of the pcDNA3-PB2-FLAG, pcDNA-PB1, 11.76 ng of pcDNA-PA, 100 ng of pcDNA3-BNP, and 100 ng of pHH21-BNA-Luc expressing plasmids. Additional plasmids were used in various amounts (NS1:50 and 75 ng; PKCD Cat:15, 30, 60, and 90 ng, respectively) and topped up by blank vectors up to 100 ng to keep the total amount of DNA same in all the sets. The transfection mixture was prepared with Lipofectamine 3,000 as stated earlier. All transfections for the luciferase activity assay have been performed in triplicate. At 12 h, the medium was changed very carefully without dislodging any cells to avoid manual variation among the sets. The cells were harvested at 36 h (or as mentioned) post-transfection, and the luciferase activity assay was performed using the Promega Luciferase Assay System (Promega, #E1500 & #E1910). Briefly, the medium was aspirated and 250 μl (1 x CCLR for firefly luciferase assay) and 100 μl (1 x PLB for dual luciferase assay) of lysis buffer (supplemented with 1 × protease inhibitor and 1x phosphatase inhibitor) was added to each well and incubated for 20 min at 4°C (firefly luciferase assay) and room temperature (dual luciferase assay), respectively. The assay was performed as per the manufacturer's protocol using a luminometer (Promega Glomax 20/20) and data were analyzed as stated in the statistical analysis section. Lysates from the three triplicate wells were pulled together for western blot analysis.

### Ribavirin and Favipiravir Dose-Response Assays in HEK 293T Cells

Ribavirin (Sigma-Aldrich, Cat no. # R9644) was dissolved in water to prepare an 80 mM stock, aliquoted and stored in a refrigerator at −20°C. Favipiravir (MedChemExpress, Cat no. #HY-14768) was dissolved in dimethyl sulfoxide (DMSO; Sigma, Cat no. #D2650) to prepare a 200 mM stock, aliquoted, and stored at −80°C until use. Working stocks for both compounds were prepared in complete media. About 0.2 × 10^6^ HEK 293T cells were seeded in 24-well plates and post 24 h were treated with specified concentrations of ribavirin or favipiravir for 2.5 h at 37°C with 5% CO_2_. Subsequently, the cells were transfected with lipofectamine 3000 as per the manufacturer's protocol and incubated in fresh media with the specified concentrations of drugs for 36 h. Polymerase activity was then assayed as described above. The IC50 value was calculated by fitting the data to a four-parameter non-linear equation.

### 3-(4,5-Dimethylthiazol-2-Yl)-2,5-Diphenyltetrazolium Bromide Assay

About 3 × 10^4^ HEK 293T cells were seeded in a 96-well plate. After 24 h of seeding, cells were treated with different concentrations of drugs in triplicate for 36 h. Post-treatment, 100 μl of 3-(4,5-dimethylthiazol-2-yl)-2,5-diphenyltetrazolium bromide (MTT) reagent [5 mg/ml, SRL, Cat no. # 33611(2049101)] dissolved in PBS was added to the cells and incubated for 3 h at 37°C. Subsequently, the reagent was removed and the formazan crystals were dissolved by adding 100 μl of DMSO to each well. The absorbance of the suspension was measured at 595 nm using an Epoch 2 microplate reader (BioTek Instruments). The percentages of drug-treated metabolically active cells were compared with the percentage of control cells treated with the vehicle control.

### Transfection-Infection Assay

About 0.1 × 10^6^ HEK 293T cells were seeded in 48-well plates. After 20 h of seeding, the cells were transfected with 0.25 μg of reporter plasmid using lipofectamine 3000 and 22-h post-transfection, the cells were pretreated with half-maximal inhibitory concentrations (IC50) of the drugs (Ribavirin:18.54 μM and Favipiravir:25.46 μM) for 2.5 h at 37°C with 5% CO_2_. Following 2.5 h of treatment, cells were infected with influenza B virus at an MOI of 0.1 in the presence of drugs and the polymerase activity was assayed at 16-h postinfection.

### Statistical Analysis

The arithmetic mean and standard deviation (SD) of the firefly luciferase signal were calculated from three biological replicates for each experiment. The mean intensity of luminescence (in arbitrary units) obtained from the luminometer was plotted in a bar diagram with SDs as error bars. For the dual luciferase assay, polymerase activity was reported as the ratio of firefly to renilla signal. In firefly luciferase assays that include the host factors, viral factors, antiviral molecules and dual luciferase assays; the normalized mean and SD were calculated against the control. The normalized mean was calculated by dividing the arithmetic mean of the experimental sets by the mean of the control set and converting them to a percentage value. The normalized SD was calculated by normalizing the coefficient of variations against the control and augmenting it with the normal mean. A two-tailed Student's *t*-test was performed to compare individual data sets. Intra-assay variability was analyzed by calculating the percentage coefficient of variation (%CV) of the different sets with biological replicates. Student's *t*-test was performed to compare the %CV of different assays. Inter-assay %CV was calculated from the mean %CV of three independent experiments.

## Results

### Generation of Influenza B Virus Reporter Plasmid for the Expression of Viral Reporter RNA in Mammalian Cells

**Figure 1:**
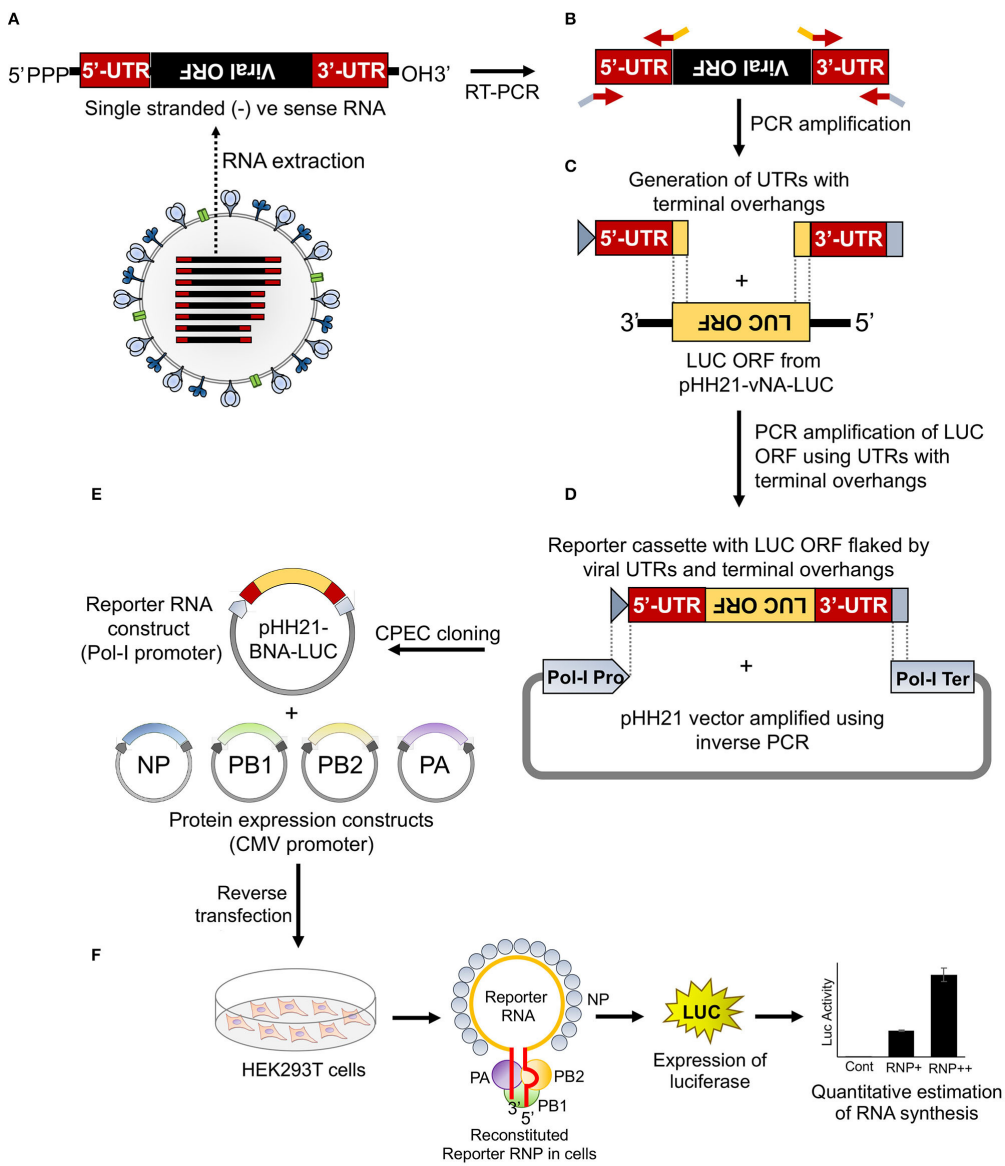
An overview of the cloning strategy of influenza B reporter plasmid and reporter assay. **(A)** Total RNA was isolated from amplified stocks of influenza B/Brisbane/60/2008 virus. **(B)** Total RNA was converted to cDNA by performing reverse transcription polymerase chain reaction (RT-PCR) and the 5′ and 3′ untranslated regions (UTRs) were amplified using specific primers containing overhangs. **(C)** The double-stranded 5′ and 3′ UTRs containing overlapping regions were used as primers to amplify the luciferase open reading frame (ORF). **(D)** The resulting PCR product was used as an insert for circular polymerase extension cloning (CPEC) assembly with the PCR amplified vector fragment. **(E,F)** The generated reporter and other protein expressing plasmids, upon co-transfection in human embryonic kidney 293T (HEK 293T) cells, reconstitute luciferase RNPs that express the luciferase enzyme under the control of the viral promoter. The quantification of the luciferase signal gives the measure of viral polymerase activity.

To establish a reporter-based RNP activity assay, a template RNA harboring the reporter gene flanked by the viral UTRs needs to be expressed under the control of RNA polymerase I promoter. This ensures that the reporter RNAs are devoid of any 5′- or 3′-terminal modifications, hence mimicking authentic viral genomic RNA. To achieve this, firstly we have constituted the “insert” harboring the firefly luciferase gene in reverse orientation flanked by the viral 5′ and 3′ UTRs. Subsequently, this cassette was introduced into the pHH21 vector in between the RNA polymerase I promoter and terminator. The entire process of constituting the authentic viral UTRs, assembling them with the reporter gene and introducing this cassette into the pHH21 vector utilized a single DNA polymerase enzyme without the need for any restriction enzyme or specialized kits (Figure 1). The sequences of 5′ UTR, 3′ UTR, and the primers corresponding to the annealing regions are depicted in Supplementary Figure S1.

**Figure 2:**
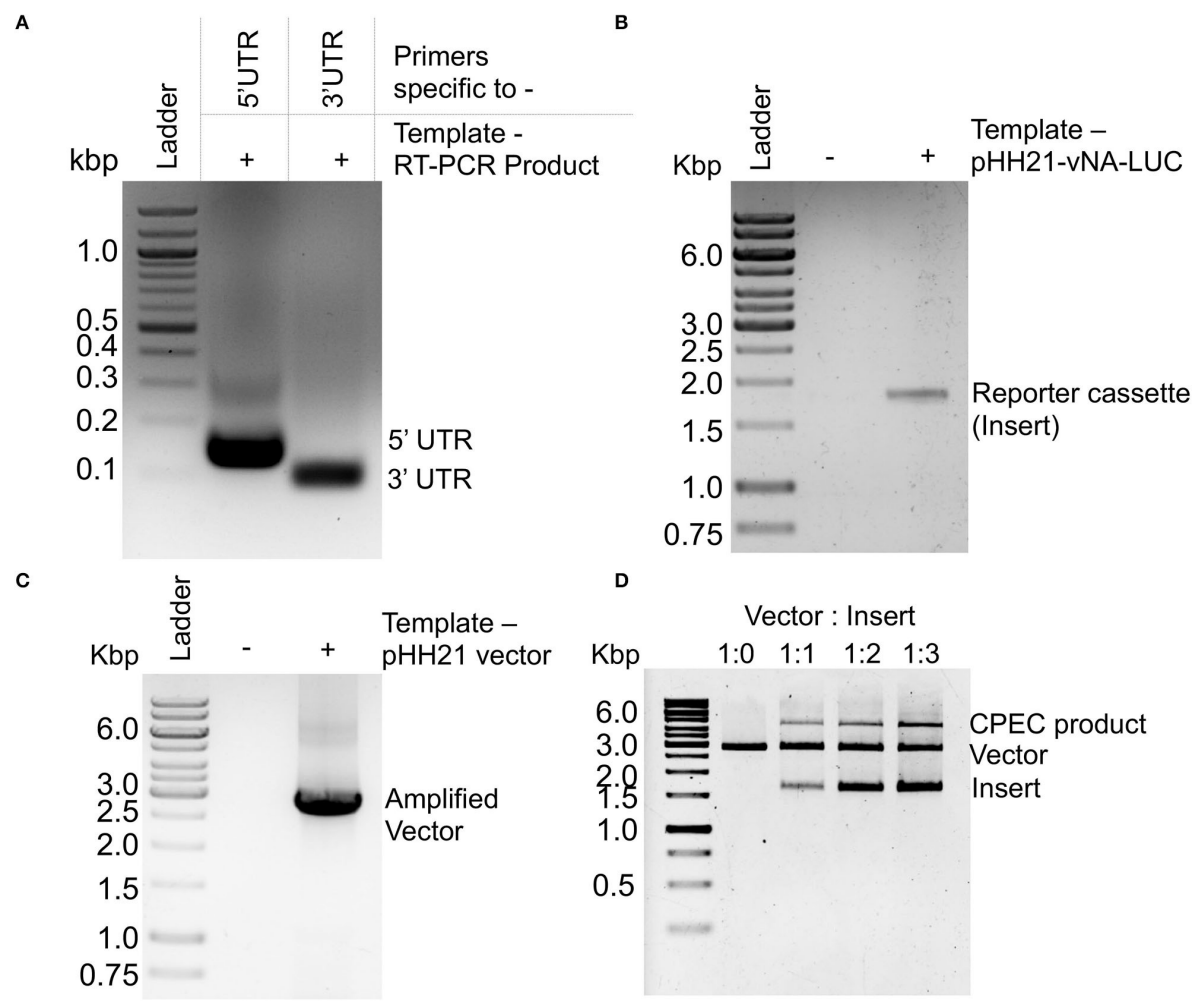
PCR amplification and CPEC reaction for the construction of a reporter construct: Agarose gel electrophoresis images of **(A)** PCR amplification products corresponding to the 5′ and 3′ UTRs of NA-NB segment. **(B)** PCR amplification product of the luciferase ORF using double-stranded PCR products corresponding to 5′ and 3′ UTRs as primers. **(C)** PCR amplification of the pHH21 vector. **(D)** CPEC products with different ratio of vector and insert.

Viral genomic RNA, purified from influenza B/Brisbane/60/2008 virus particles (as depicted in Figure 1A), was used as a source for the amplification of 5′ and 3′ UTRs (103 and 53 nucleotides, respectively), using sequence-specific primers with 18–24 nucleotide overhanging sequences corresponding to the vector and the reporter gene (Figures 1B, 2A). The resulting PCR products thus contain (i) the viral 5′-UTR flanked by the overlapping sequences with the Pol-I promoter and 3′-termini of the reporter gene of a total of 144 base pairs in length and (ii) the viral 3′-UTR region flanked by the overlapping sequence with the 5′-termini of the reporter gene and Pol-I terminator of a total of 95 base pairs in length. These double-stranded PCR products were then used as primers to amplify the firefly luciferase gene from the pHH21-vNA-Luc plasmid, kindly provided by Prof. Andrew Mehle, University of Wisconsin Madison (Figures 1C, 2B). The final PCR product constitutes the reporter gene flanked by the viral 5′- and 3′-UTR regions along with the partial sequences from the Pol-I promoter and terminator regions at the extreme 5′ and 3′ termini, respectively. To synthesize the final reporter plasmid construct, named as pHH21-BNA-Luc, this cassette was inserted into the pHH21 vector (amplified in a separate PCR reaction; Figure 2C) using the CPEC, as originally described by Quan and Tian (2009, 2011) (outlined in Figure 1D). A vector to insert molar ratio of 1:3 generated the maximum amount of the assembled product (Figure 2D). The reaction product was transformed into chemically competent *E. coli*, and the successful incorporation of the insert was confirmed by the colony PCR screening method. All the PCR amplifications were performed using a single Phusion high-fidelity DNA polymerase, as described in further detail in Section Materials and Methods.

To reconstitute functional reporter RNPs inside the cells, the reporter RNA template needs to be co-expressed with NP and the RdRp subunits, PB1, PB2, and PA (Figures 1E,F). The RdRp subunits were cloned into the pCDNA-3 × -FLAG vector (generously provided by Dr. Andrew Mehle) under the control of the CMV promoter with the help of EcoRI and NotI restriction enzymes, which results in the incorporation of a tri-alanine linker between the individual ORFs and the three FLAG epitopes joined in tandem (3 × -FLAG). For the expression of untagged proteins, ORFs with the stop codon were cloned using the same strategy. The NP gene was cloned into a modified pcDNA3 vector harboring the V5 epitope tag (as mentioned in Section Materials and Methods) with the help of KpnI and BamHI sites, with a glycine–glycine–serine–glycine linker in between the ORF and the V5 epitope tag.

### Standardization of Influenza B Virus RNP Activity Assay

**Figure 3:**
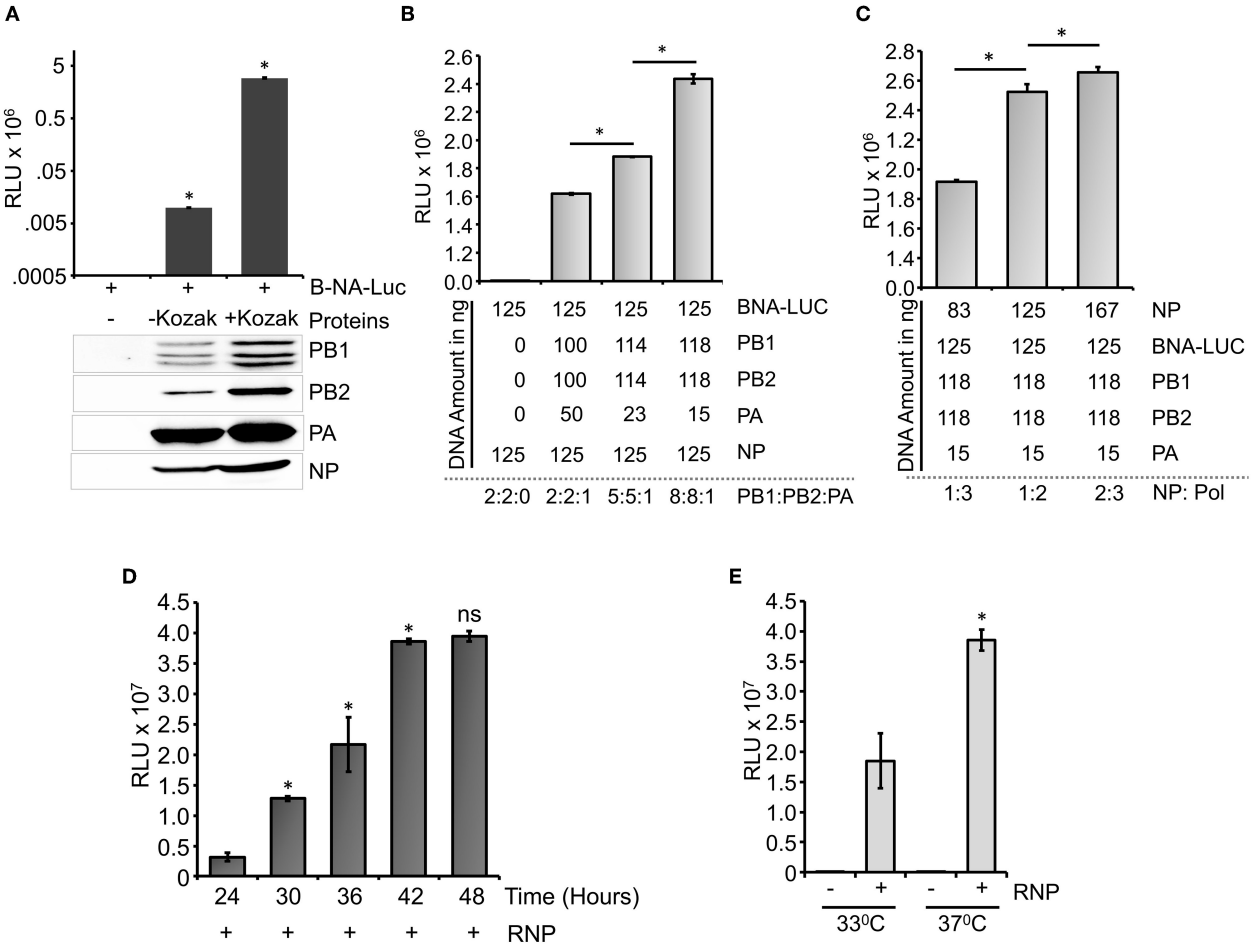
Optimization of the reporter system: **(A)** An effect of Kozak sequence on the expression of viral polymerase proteins and influenza B RNP activity assay. **(B)** Reporter RNP activity assay with different ratios of the PA protein expression plasmid with respect to PB1 and PB2. **(C)** Reporter RNP activity assay with various amounts of NP expression plasmid. **(D)** Optimization of time for the reporter activity assay. **(E)** Optimization of incubation temperature for the influenza B RNP activity assay (*n* = 3 ± standard deviation (SD), ^*^p < 0.05 one-way analysis of variance (ANOVA) with *post-hoc* Student's *t*-test when compared to the preceding set, for **(E)**, a comparison was performed between two RNP positive sets, ns, not significant).

Influenza B virus reporter RNPs were reconstituted in HEK 293T cells through transient transfection of the reporter plasmid (pHH21-BNA-Luc) either in the absence (negative control) or presence of plasmids expressing PB1, PB2, PA, and NP proteins (Figure 3A). Cells were harvested at 24-h post-transfection, and luciferase activity was measured to quantify influenza B RNP activity. To our surprise, the set of positive controls only showed a signal of ~10^4^ (luciferase light unit or RLU), which is only two log higher than that of the set of negative controls (~10^2^ RLU), suggesting suboptimal activity of the reconstituted RNPs. This could be due to the poor expression levels of the RdRp subunits, PB1 and PB2, in comparison to the PA and NP proteins, as observed by the western blot analysis using specific antibodies, hence prohibiting the successful assembly of reporter RNPs inside the cells. We analyzed the expression of individual polymerase proteins, PB2, PB1, and PA by transfecting them in increasing amounts into HEK 293T cells and subsequent western blot analysis, as shown in Supplementary Figure S2. It was observed that the expression of PA subunit is significantly higher than PB2 and PB1. To investigate this further, we examined the sequences of the constructs carefully and noticed that all protein expression plasmids lack the upstream Kozak sequence, which may result in their suboptimal translation. The conserved Kozak sequence (GCCRCCATGG) plays a critical role in the recognition of an initiator ATG codon by the ribosome to attain a high level of translation. Two specific positions, −3 and +4 from the adenine of the initiator codon ATG are found (GCC**R**CCATG**G**) to be critical for the optimal protein expression (Kozak, 1986, 1987, 1997; Sakai et al., 2001). The PA gene has a Guanosine at the +4 position in its ORF but PB1 and PB2 do not have this Guanosine in their ORF. This makes the expression of PA gene better than that of PB2 and PB1. To address this, we performed site-directed mutagenesis to introduce partial Kozak sequences into each of these plasmids without any alteration in the ORF and repeated the polymerase activity assay with them. As evident from Figure 3A, the introduction of the Kozak sequence significantly boosted the expression of all RNP proteins, which together resulted in a reporter activity of 10^6^ RLU, four logarithms higher than the negative control set. Interestingly, the expression levels of the PA subunit still remained severalfold higher than the other two subunits of RdRp, PB1, and PB2 (Figure 3A). Precise abundance of the PB1, PB2, and PA subunits in equimolar amounts is a prerequisite for the successful assembly of the heterotrimeric RdRp complex and hence reconstitution of reporter RNPs to optimum levels. Therefore, we tried to optimize the amount of the plasmids to be transfected to have a comparable expression of the RdRp subunits. Reporter RNPs were reconstituted using different ratios of RdRp subunit plasmids, while keeping the amount of the reporter RNA and NP plasmids constant. As shown in Figure 3B, increasing the amount of PB1 and PB2 expressing plasmids compared to PA led to a gradual increase in reporter activity and a ratio of 8:8:1 for PB1: PB2: PA resulted in comparable expression of all three polymerase subunits and maximal reporter activity. Protein expression level for all polymerase subunits has been shown by western blot analysis in Supplementary Figure S3. Subsequently, keeping the ratio of the polymerase subunit plasmids constant, we increased the amount of the NP expressing plasmid, which resulted in increase in the reporter activity, hence stretching the sensitivity of this reporter assay to the maximum level (Figure 3C). Individual protein expression levels are shown in Supplementary Figure S4 as western blot analysis. The NP to polymerase proportion up to 1:2 results in an increase in polymerase activity. Further increase in the amount of NP does not result in a substantial increase in polymerase activity. Hence, for our further experiments, we have used this ratio of RNP reconstituting plasmids. Once we optimize the amounts of various plasmids that reconstitute reporter RNPs, we have performed a time kinetics experiment to assess the optimal time required to obtain a signal of 10^6^ RLU or more (Figure 3D).

A time-dependent increase in the reporter activity was observed which reaches a plateau at 42 h post-transfection. Additionally, influenza B polymerase activity was assessed at different temperatures (33°C and 37°C) by reconstituting the polymerase through transient transfection at 37°C for 12 h followed by an additional incubation of 30 h at respective temperatures (Figure 3E). As observed at 37°C, the reporter activity was almost 2-fold higher than the activity at 33°C, a data corroborated perfectly with previous results obtained by Santos et al. (2017). Taken together, we present a fast, sensitive, and high-throughput reporter assay for monitoring influenza B virus RNA synthesis in an infection-free setting.

All of the assays were performed in 24-, 48-, and 96-well plates, in triplicate for each of the biological sets (data presented in this manuscript is from 24-well plates), hence confirming that this assay system is high-throughput compatible. Additionally, five log difference between the signal and background readouts provides a wide dynamic range for this reporter-based assay system. There is no significant difference in the intra-assay variability among individual experiments, as shown in Supplementary Figure S5. The inter-assay coefficient of variation remains within 10% (Supplementary Figure S5). Taken together, we have been able to establish a fast, reliable, and high-throughput compatible assay system for monitoring influenza B virus RNP/ polymerase activity, which is suitable for assessing the effect of various viral or cellular factors in modulating RNP activity and hence viral RNA synthesis.

### The Influenza B RNP Activity Assay Is Suitable for Evaluating the Efficacy of Viral or Host Factors in Regulating Viral RNA Synthesis

**Figure 4:**
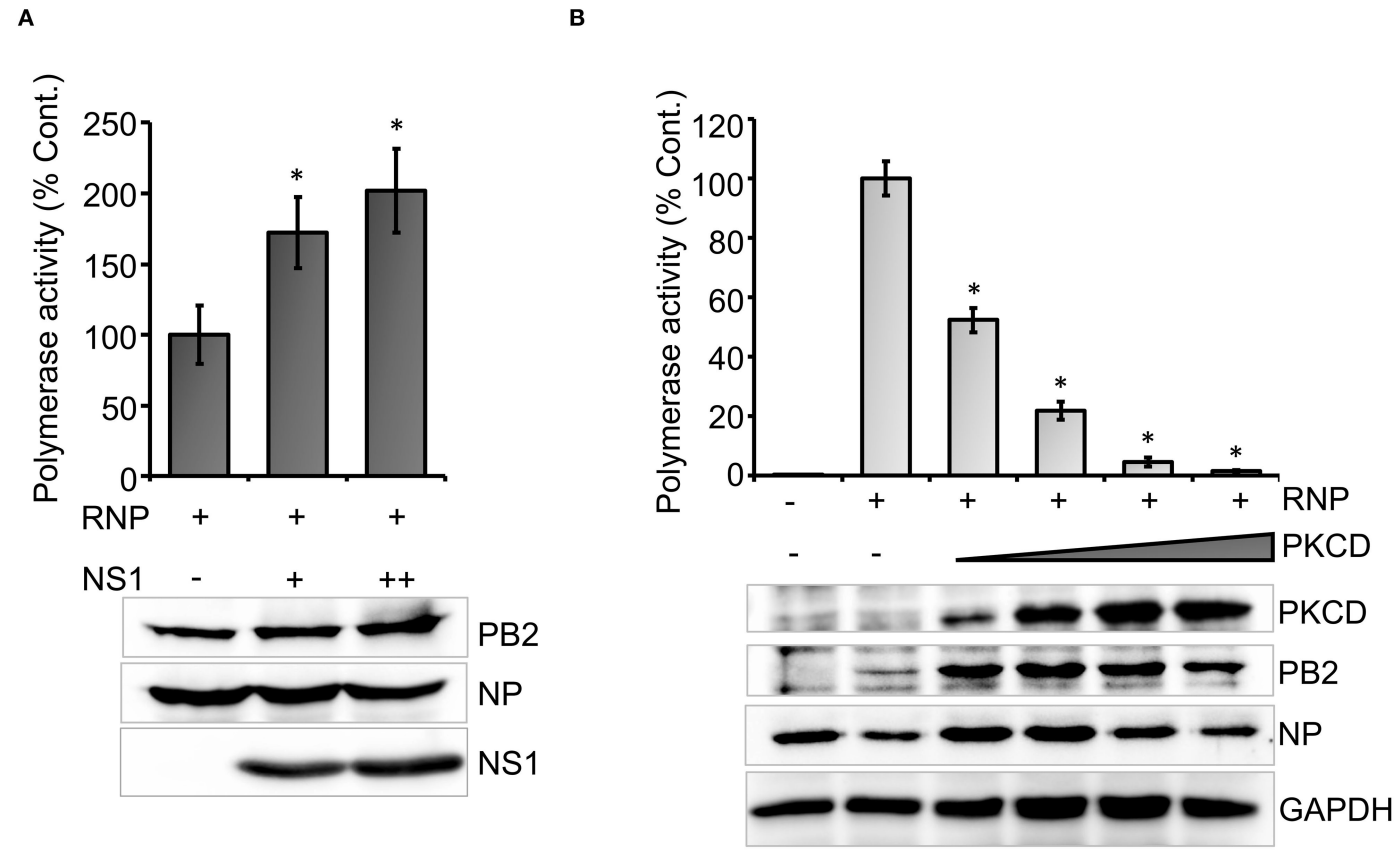
An effect of host and viral factors upon viral RNA synthesis: **(A)** effect of an increasing amount of viral NS1 protein on influenza B RNP activity assay and **(B)** effect of an increasing amount of constitutively active host protein kinase C delta (PKCD) protein on B RNP activity assay (*n* = 3 ± SD. ^*^p < 0.05 one-way ANOVA with *post-hoc* student's *t*-test when compared to the preceding set.

To this end, we set out to evaluate the efficacy of the newly developed polymerase activity assay to identify novel viral and host factors that may regulate viral RNA synthesis. Influenza virus Non-structural protein-1 (NS1) is a multifunctional protein that participates mainly in the suppression of antiviral defense mechanisms exerted by a wide variety of host factors (Tisoncik et al., 2011; Nogales et al., 2019). Additionally, influenza A virus NS1 protein has been shown to boost viral RNA synthesis (Salvatore et al., 2002; Mahmoudian et al., 2009; Huang et al., 2017), possibly through interference with the antiviral activity of DDX21 and RAP55 (Mok et al., 2012; Chen et al., 2014). While the immune suppression activity of influenza B NS1 has been well-studied (Yuan and Krug, 2001; Dauber et al., 2004; Nogales et al., 2019), little is known about the role of NS1 in regulating influenza B virus RNA synthesis. Hence, we evaluated the ability of the influenza B virus NS1 protein to promote viral RNA synthesis with the newly developed reporter RNP activity assay. Influenza B virus NS1 ORF was cloned into the pCDNA-3 × -FLAG vector, which resulted in the expression of the C-terminal FLAG-tagged NS1 protein. Influenza B reporter RNPs were reconstituted in HEK 293T cells either in the absence or presence of increasing amounts of NS1 protein, and reporter activity was monitored to assess the extent of viral RNA synthesis. Increasing the amount of NS1 resulted in a 1.5- to 2-fold increase in reporter activity (Figure 4A), establishing that it as a positive regulator of viral RNA synthesis. Furthermore, reconstitution of reporter RNPs in the presence of NS1 presents an assay system that closely resembles RNA synthesis, which occurs during the course of infection.

Subsequently, we tested the ability of a host factor to regulate influenza B virus RNA synthesis using our reporter RNP activity assay. Host protein kinase C (PKC), specifically the delta isoform, has been shown to positively influence influenza A virus RNA synthesis by regulating the phosphorylation and subsequent assembly of viral nucleoproteins into RNPs. Interestingly, the constitutively active catalytic domain of PKCD, when overexpressed, negatively regulates influenza A virus RNA synthesis (Mondal et al., 2017). To determine the role of PKCD in regulating influenza B virus RNA synthesis, we employed the newly developed reporter RNP activity assay. As evident from Figure 4B, increasing amounts of PKCD resulted in a gradual decrease in RNP activity and hence viral RNA synthesis, without any severe impact upon the translation of viral proteins. These data not only substantiate the role of PKCD in regulating influenza B virus RNP activity, but also validate the efficacy of our assay system to study the effect of proviral or antiviral factors regulating viral RNA synthesis.

### Reporter-Based RNP Activity Assay as High-Throughput Screening Platform for Antiviral Drugs

**Figure 5:**
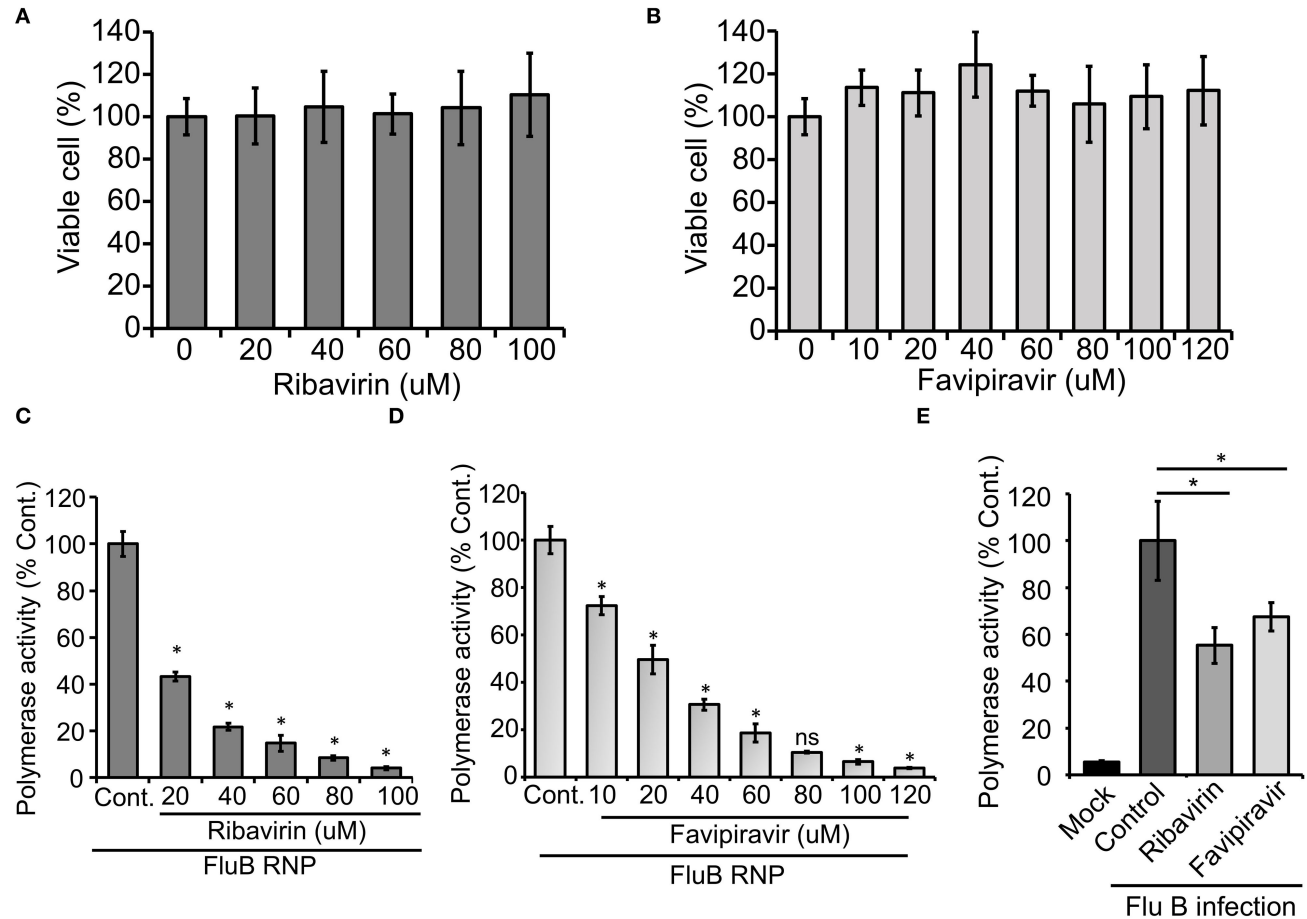
An effect of antiviral drugs upon viral RNA synthesis in an infection-free and infection setting: **(A,B)** 3-(4,5-dimethylthiazol-2-yl)-2,5-diphenyltetrazolium bromide (MTT) assay to determine the cytotoxicity of Ribavirin and Favipiravir on HEK 293T cells. **(C–E)** The effect of Ribavirin and Favipiravir on influenza B virus RNP activity. Viral polymerase proteins in HEK 293T cells are expressed by transient transfection **(C,D)** or by infecting the cells with influenza B virus **(E)** (*n* = 3 ± SD, ^*^p < 0.05 one-way ANOVA with *post-hoc* Student's *t*-test when compared to the preceding set, for **(E)**, comparison was performed with control set, ns, not significant).

Finally, we intend to establish the suitability of the RNP activity assay as a high-throughput screening platform for antiviral drugs that can inhibit viral RNA synthesis and hence virus replication. Ribavirin and Favipiravir are nucleoside (purine) analogs, which inhibit the replication of a wide variety of RNA viruses by acting as an alternative substrate for viral RNA polymerase (Graci and Cameron, 2006; Furuta et al., 2017). Additionally, Ribavirin also inhibits inosine monophosphate dehydrogenase, thereby depleting the GTP and creating an imbalance in the nucleotide pool inside the cell (Gish, 2006). Both Ribavirin and Favipiravir have been approved as chemoprophylaxis as well as therapy against influenza A and B viruses (Oxford, 1975; McCracken and McCracken, 1976; Cheung et al., 2014). Hence, we used these two drugs as positive controls to test the efficacy of our assay system for antiviral screening. The MTT assay was performed in HEK 293T cells (Figures 5A,B), where neither of the drugs show any cytotoxicity up to the concentrations investigated. HEK 293T cells were pretreated with different concentrations of the drugs, followed by forward transfection to reconstitute influenza B reporter RNPs and subsequent incubation with the drugs for 36 h. Reporter activities were measured and expressed as relative percentages with respect to the vehicle control. The data presented in Figures 5C,D, show a dose-dependent decrease in reporter activity and hence viral RNA synthesis with increasing amounts of the drugs with IC50 values of 18.54 μM and 25.46 μM for Ribavirin and Favipiravir, respectively.

To further extend the scope of the assay system, we sought to check if this system is capable of assessing the effect of host factors or antivirals on the overall progress of infection. For this purpose, we transfected HEK 293T cells with the reporter construct and subsequently infected them with influenza B virus at 20 h post-transfection. It is expected that, in infected cells, the reporter RNA template will get transcribed with the help of RdRp and NP proteins expressed from viral genomic RNA segments. As evidenced in Figure 5E, infected cells supported successful generation of reporter RNPs and hence showed high reporter activity, while uninfected cells showed no such effects. Interestingly, when a parallel set of cells were treated with Ribavirin and Favipiravir prior to infection with influenza B virus, a significant reduction in reporter activity was observed, hence suggesting an overall reduction in viral gene expression and hence virus replication in the presence of the drugs. Taken together, our data reconfirm the activity of the two well-established antiviral drugs against influenza B virus RNA synthesis machinery and also establishes the newly developed reporter-based influenza B virus RNP activity assay as a high-throughput screening platform for antivirals that specifically inhibit viral RNA synthesis.

## Discussion

Luciferase-based reporter assay systems remain a key tool for analyzing gene expression in a wide variety of organisms; viruses are no exceptions. Meanwhile, for positive-sense RNA viruses, the introduction of the single sub-genomic reporter RNA template into cells is sufficient for the expression of reporter genes, but for negative-sense RNA viruses, RNP-associated viral proteins need to be synthesized along with the reporter RNA to reconstitute complete RNPs, which then leads to the expression of the reporter enzyme as a proxy of viral genes (Lutz et al., 2005; Su et al., 2015; Ren et al., 2016). This is why, successful reconstruction of reporter viral RNPs require extensive cloning of multiple RNA and protein expressing constructs, the standardization of their expression in correct stoichiometric ratios, and the optimization of other crucial parameters like time, temperature, etc. Although several groups have reported reporter assay systems for monitoring the RNA synthesis of influenza A and B viruses, non-availability of a detailed methodical description makes the process of establishing the assay system non-trivial (Lutz et al., 2005; Wang and Duke, 2007; Li et al., 2009; Zhu et al., 2011; Moncorge et al., 2013). In this work, we have established a firefly luciferase-based influenza B virus RNP activity assay and presented the detailed methodology of the entire procedure, which could be easily followed for the development of such viral and non-viral reporter assay systems.

We have introduced a unique cloning strategy for the construction of the influenza B virus reporter RNA construct that is devoid of restriction enzymes or any other specialized enzymes. This cloning strategy utilizes a single DNA polymerase, which is widely used for regular molecular biology work and hence easily available. Using this polymerase, two consecutive PCR amplification reactions led to the generation of the reporter RNA cassette encompassing the reporter ORF flanked by the viral 5′- and 3′-UTR regions which were then inserted into the vector using the CPEC cloning method. While the vector and the insert used for CPEC are also compatible for Gibson assembly-based cloning method, we intentionally avoided the use of any specialized enzymes to make the overall procedure simple, user-friendly, and potentially being adapted for the cloning of any other reporter RNA constructs. In addition to the reporter RNA construct, we also cloned ORFs corresponding to the viral proteins PB1, PB2, PA, and NP and optimized their expression to reconstitute reporter RNPs at maximum levels. The robustness of this assay system was substantiated by testing the efficacy of the antiviral drugs, Ribavirin and Favipiravir, to inhibit influenza B virus RNA synthesis either in the context of reconstituted RNPs (through transfection) and during the course of infection. The fact that the reporter RNA template can be preferentially recognized by the viral NP and RdRp subunits to reconstitute reporter RNPs during the course of infection confirms that the reporter RNA mimics viral genomic RNA segments and hence validates its suitability for use in the study of viral RNA synthesis and the effect of various viral and host factors on it. In fact, using the newly developed reporter RNP system, we showed for the first time that viral NS1 protein can boost influenza B virus RNA synthesis and the constitutively active form of host PKCD can downregulate it. While the effect of NS1 and PKCD proteins has been previously characterized in case of influenza A virus (Salvatore et al., 2002; Mahmoudian et al., 2009; Huang et al., 2017; Mondal et al., 2017), our results substantiate that these proteins participate similarly in the regulation of influenza B virus RNA synthesis as well.

Altogether, we present a comprehensive roadmap for the development, characterization, and validation of a reporter-based Influenza B virus polymerase/RNP activity assay and made it generic enough to be followed by others who intend to develop similar assay systems for influenza and other negative-sense RNA viruses. We also made all the resources publicly available (upon request) to enrich the armory for combating influenza B viruses and hope that it will be widely utilized to identify new therapeutic strategies against this deadly human pathogen.

## Data Availability Statement

The original contributions presented in the study are included in the article/Supplementary Material, further inquiries can be directed to the corresponding author/s.

## Author Contributions

NK designed and performed the experiment, standardized methodology, analysed the data, wrote the original draft, and reviewed and edited the manuscript. SB designed and performed experiments, analyzed the data, and reviewed and edited the manuscript. AM conceptualized the work, designed and supervised the project, arranged for funds, wrote the original draft, and reviewed and edited the manuscript. All authors have read and approved this final manuscript.

## Funding

Financial support from the following sources is gratefully acknowledged. DBT Ramalingaswami re-entry fellowship (BT/RLF/Re-entry/02/2015), Department of Biotechnology, Government of India; DST-SERB, Early Career Research Award (ECR/2017/001896), Science and Engineering Research Board, Department of Science and Technology, Government of India; and Scheme for Transformational and Advanced Research in Science {STARS/APR2019/BS/369/FS (Project ID: 369)}, Ministry of Education, Government of India.

## Conflict of Interest

The authors declare that the research was conducted in the absence of any commercial or financial relationships that could be construed as a potential conflict of interest.

## Publisher's Note

All claims expressed in this article are solely those of the authors and do not necessarily represent those of their affiliated organizations, or those of the publisher, the editors and the reviewers. Any product that may be evaluated in this article, or claim that may be made by its manufacturer, is not guaranteed or endorsed by the publisher.
